# Highly varying concepts and capacities of forensic mental health services across the European Union

**DOI:** 10.3389/fpubh.2023.1095743

**Published:** 2023-01-26

**Authors:** Hans Joachim Salize, Harald Dressing, Heiner Fangerau, Pawel Gosek, Janusz Heitzman, Inga Markiewicz, Andreas Meyer-Lindenberg, Thomas Stompe, Johannes Wancata, Marco Piccioni, Giovanni de Girolamo

**Affiliations:** ^1^Central Institute of Mental Health Mannheim, Medical Faculty Mannheim, Heidelberg University, Mannheim, Germany; ^2^Institute of History, Theory and Ethics in Medicine, University Hospital Düsseldorf, Dusseldorf, Germany; ^3^Department of Forensic Psychiatry, Institute of Psychiatry and Neurology, Warsaw, Poland; ^4^Clinical Division of Social Psychiatry, Medical University of Vienna, Vienna, Austria; ^5^Forensic Psychiatric Hospital, Göllersdorf, Austria; ^6^Department of Forensic and Neurodevelopmental Science, Institute of Psychiatry, Psychology and Neuroscience, King's College London, London, United Kingdom; ^7^St. Magnus Hospital, Haslemere, United Kingdom; ^8^Istituto di Ricovero e Cura a Carattere Scientifico (IRCCS) Istituto Centro San Giovanni di Dio Fatebenefratelli, Brescia, Italy

**Keywords:** forensic psychiatric care, mentally ill offenders, forensic psychiatric prevalence, forensic psychiatric incidence, mental health policies

## Abstract

**Introduction:**

There is wide variation in the processes, structures and treatment models for dealing with mentally disordered offenders across the European Union. There is a serious lack of data on population levels of need, national service capacities, or treatment outcome. This prevents us from comparing the different management and treatment approaches internationally and from identifying models of good practice and indeed what represents financial efficiency, in a sector that is universally needed.

**Methods:**

From March 2019 till January 2020 we surveyed forensic psychiatric experts from each European Union Member State on basic concepts, service capacities and indicators for the prevalence and incidence of various forensic psychiatric system components. Each expert completed a detailed questionnaire for their respective country using the best available data.

**Results:**

Finally, 22 EU Member States and Switzerland participated in the survey. Due to the frequent lack of a clear definition of what represented a forensic psychiatric bed, exact numbers on bed availability across specialized forensic hospitals or wards, general psychiatric hospitals or prison medical wards were often unknown or could only be estimated in a number of countries. Population-based rates calculated from the survey data suggested a highly variable pattern of forensic psychiatric provision across Europe, ranging from 0.9 forensic psychiatric beds per 100,000 population in Italy to 23.3 in Belgium. Other key service characteristics were similarly heterogeneous.

**Discussion:**

Our results show that systems for detaining and treating mentally disordered offenders are highly diverse across European Union Member States. Systems appear to have been designed and reformed with insufficient evidence. Service designers, managers and health care planners in this field lack the most basic of information to describe their systems and analyse their outcomes. As a basic, minimum standardized national reporting systems must be implemented to inform regular EU wide forensic psychiatry reports as a prerequisite to allow the evaluation and comparison of the various systems to identify models of best practice, effectiveness and efficiency.

## Introduction

The most appropriate location and treatment of people who commit offenses as a result of or while suffering from a mental disorder is an important consideration for societies and nations globally. A key challenge is to ethically implement basic principles such as that is only offenders who are responsible for their actions should be punished. Mentally disordered offenders who remain dangerous should be taken into some form of custody and treated to reduce the risk of further offending.

Internationally, a wide variety of judicial, medical and organizational approaches have evolved for this. Institutions and services where mentally ill offenders are detained and treated usually are labeled as “forensic psychiatry.” The wide-spread usage of this term suggests an international consensus regarding standards and processes for dealing with mentally disordered offenders. However, that is not the case. Guidelines for managing and treating forensic populations are scarce and typically quite general. Models of good practice are not agreed (1–4). Thus, this common label tends to conceal huge differences of the structure, size, organization and budgets of national forensic psychiatric systems.

Solid data, international evidence or essential knowledge from this sector is lacking, however. There are only a few studies on these issues available from the past, that covered only a small number of selected countries (5–9). These studies more or less used similar methods for assessing bed-rates or other data as applied in this study here, either by expert rating or by referring to administrative data, which usually turned out to be scarcely available, incomplete or of limited validity and reliability. The results suggest a wide variety in basic indicators, concepts and models.

This situation increases the risk that changes to the services for mentally disordered offenders are not triggered by clinical or legal needs but by largely extraneous economic or political considerations. Italy provides the most recent example in this when it recently closed its six established forensic psychiatric hospitals and replaced them with small community residential facilities (10, 11). However, this fundamental change had no international examples to guide them. The reorganization was not based on research if the new approach would lead to better treatment outcomes, more or fewer restrictions or how they would harm patient or public safety (12).

Initiatives as the EU-funded COST-Action IS1302 “Toward an EU research framework on Forensic Psychiatric care” launched in 2013 (9) or the so-called Ghent group (13), that try to intensify European research activities have criticized together with other experts in the field the lack of research evidence to inform the planning and design of forensic psychiatric services (4, 6, 14–16).

Research in this field faces considerable methodological challenges given that there is no clear consensus even on what a “forensic psychiatric bed” is or whether such beds should be in specialized forensic psychiatric hospitals, medical wards in prisons or in general psychiatric settings.

In order to address some of these problems, an EU-wide survey of the concepts and capacities for placing and treating mentally disordered offenders was conducted between 2018 and 2020 (with March 2019 as a starting point for the actual data collection). The survey was a part of the multi-center “European Study on Risk Factors for Violence in Mental Disorder and Forensic Care (EU-VIORMED)” (17).

## Materials and methods

The survey was designed and managed from the Central Institute of Mental Health in Mannheim, Germany. It aimed to collect data from national forensic psychiatric experts in all European Union Member States. These experts were supposed to fill in a semi-structured questionnaire, adapted from the survey instrument used in a similar EU-study between 2003 and 2005 (6, 18). The questionnaire included 52 questions across 9 sections that covered: forensic psychiatry legislation, key concepts, forensic psychiatry assessment, court and trial procedures, placement and treatment procedures, re-assessment and discharge, patients' rights, service provision and epidemiology (indicators of prevalence and incidence of forensic psychiatric patients, mean length of stay etc.). All indicators and items of the questionnaire were defined and standardized as precisely as possible to prevent methodological uncertainty and assure comparability between nations and systems. The questionnaire asked for the most recent data available for all items. For more detailed information see the original questionnaire in the supplement material of this paper.

A list of potential collaborators and experts from every EU Member States was compiled initially. These were contacted and invited to collaborate from July 2018 onwards. After agreement, the experts were subcontracted to the study and received a small allowance. All collaborators were proven experts in the field of forensic psychiatry in their country, most of them being long-term members of the COST-Action IS 1302 or the Ghent Group (see above) or being otherwise involved into international research or having administrative responsibilities for forensic psychiatric issues of their country. The questionnaires (always in English and not translated into the local languages of the respective countries) were distributed by e-mail with an initial deadline for return (by mail) by April 2019. Apart from specifications and definitions of items in the questionnaire, the experts did not receive training to complete the survey. After extending the deadline in some cases the last questionnaire was returned in January 2020, after which the survey was declared as closed. Although being invited and included into the original sample, experts from Greece, Hungary, Lithuania, Malta, the Netherlands and Slovakia did not reply or return the questionnaire even after deadline extension. Thus, the final sample included experts from 22 countries and Switzerland, a non-EU Member State. From the UK, England was represented. This paper presents the principle survey results regarding indicators for forensic psychiatric service provision (e.g., number of beds, prevalence etc.).

### Data analysis

According to the major aim of the survey, data analysis methods were mostly descriptive. Due to incompleteness and limited validity or reliability of data or concepts, statistical power was not given and statistical tests were not applied, particularly not in the few cases where time series were reported.

Due to uncertainty and heterogeneity of concepts internationally in use we defined indicators as shown in Table 1. If survey data was associated to external data or indicators, official data sources were used, such as EUROSTAT (e.g., population figures for the respective countries and years). Basically, national level data was asked for and included into the comparison. A few cases of regional instead of nationwide coverage are marked.

**Table 1:** Indicators and estimates used in this study.

**Indicator or estimate**	**Definition**
Beds for forensic psychiatric patients	Number of beds in specialized forensic in- and outpatient services, general psychiatric hospitals and medical prison wards with a specific assignment or are officially registered as bed or place for the treatment and/or detention of forensic psychiatric patients or mentally ill offenders (criteria and assignment procedures may differ across countries). If official number is unknown, estimated figures were included (marked)
Rate of forensic psychiatric beds per country	Beds for forensic psychiatric patients (as defined above) per 100,000 population [population figures of countries by Eurostat (19)]
Estimated treated prevalence rate of mentally ill offenders	a) Number of forensic psychiatric patients in specialized forensic psychiatric facilities (when reported) per 100,000 population b) Number of forensic psychiatric patients in all facilities (when reported) per 100,000 population [population figures of countries by Eurostat (19)]
Indicators of incidence of mentally ill offenders	Annual number of verdicts for defendants found not being guilty by reason of insanity per 100,000 population and year [annual population number by Eurostat (19)]

## Results

### Legal frameworks

The basic legal framework governing forensic psychiatric issues between primarily health or criminal laws might give some indication of how each nation considers the detention and treatment of mentally disordered offenders. It exemplifies whether this issue is seen primarily as a matter of clinical need or public safety. According to the information provided, England and Finland regulate the placement and treatment of mentally disordered offenders by their mental or public health acts. In Austria, Belgium, Latvia, Poland, Portugal, Slovenia, Spain and Switzerland the judicial framework is predominately governed through the national criminal laws. In other countries, such as Bulgaria, Croatia, Cyprus, the Czech Republic, Denmark, Estonia, France, Germany, Ireland, Italy, Luxembourg, Romania and Sweden the regulations are spread across penal/criminal laws and mental health or public health acts. In federally organized countries such as Germany, the legal frameworks vary regionally and so cannot be characterized nationally. The majority of European laws and regulations cover the full range of ICD or DSM mental disorders. However, the laws often fail to specify diagnostic categories, prognostic criteria or definitions of disorders that are included, leaving it open to judicial practice to include all mental disorders.

### Service provision

Generally, mentally disordered offenders may be detained and treated in a wide variety of service types and facilities in both health and penal settings. These include specialized forensic in- and outpatient services, general psychiatric hospitals and medical prison wards. Table 2 shows the estimated number of forensic psychiatric beds or places provided across the European Union in 2017 as reported during the survey.

**Table 2:** Number of defined forensic psychiatric beds in European Union Member States in 2017 (empty cells: no answer or unknown).

**Country**	**Population (total number)**	**Beds in specialized forensic facilities**	**Places in forensic outpatient services**	**Forensic beds in general psychiatric facilities**	**Forensic beds in prison services**	**Forensic beds in other services**
Austria	8,772,900	440	950	210	230	
Belgium	11,365,800	891	1,110	1,068	694	
Bulgaria	7,101,000	25		350		
Croatia	4,154,200	351	4	0	40	
Cyprus	854,800				10	
Czech Republic	10,578,800	316	1,031		20	
Denmark	5,748,800	391		221		
England	55,286,100	6,590		0		0
Estonia	1,315,600	110				
Finland	5,503,300					
France	67,024,500	656			800	
Germany	82,800,000	10,799				
Ireland	4,774,800	104	21		262	
Italy	60,589,400	541				
Latvia	1,950,100	0	0	45		0
Luxembourg	590,700	42				
Poland	37,973,000	2,631	0	0	253	65
Portugal	10,309,600	275	0	0	0	
Romania	19,638,300	1,070			149	
Slovenia	2,065,900	48				
Spain	46,529,000				626	
Sweden	9,995,200	1,159	804			
Switzerland	8,417,700					543

Data origin and range of capacities as provided by experts during the survey.

Austria: Specialized forensic facilities: Ministry of Justice, forensic outpatient services: Pro Mente report.

Belgium: Psychiatric beds in prison services: Office of the Director General for Correctional Facilities, annual reports 2012–2018, annual average of patients per day in psychiatric prison wards.

Bulgaria: Sources not specified.

Croatia: Sources not specified.

Cyprus: Sources not specified.

Czech Republic: Sources not specified.

Denmark: Specialized forensic facilities: Benchmarking af psykiatrien for 2017, forensic outpatient services: Not specified. The Census of Danish Regions reported 772 forensic psychiatric outpatients in 2010, however, forensic beds in general psychiatric hospitals: not specified, The Census of Danish Regions reported 221 forensic psychiatric inpatients in general psychiatric hospitals in 2010.

Estonia: Specialized forensic facilities: Source not specified, forensic outpatient services: Provided, number of places unknown.

Finland: Numbers unknown.

France: Specialized forensic facilities: Beds in “Unités pour maladies difficiles UMD” are both for mentally ill offenders and for non-offending, but dangerous mentally ill persons. Forensic beds in general psychiatric facilities: Beds for mentally ill offenders available in unknown number, as these services do not distinguish between of involuntarily placed mentally ill and mentally ill offenders. Forensic beds in prison services: provided for psychiatric or psychological treatment of criminal fully responsible inmates also.

Germany: Specialized forensic facilities: Federal Statistical Office: Maßregelvollzugsstatistik: Number of occupied beds at January 1, 2017 in 11 out of 16 federal states, incl. Patients under § 126a.

Italy: Specialized forensic facilities (REMS): Corleone F (2019) Relazione smestrale sull'attività svolta dal Commissario unico per il superamento degli Ospedali Pschiatrici Giudiziari.

Latvia: General psychiatric services: source not specified.

Luxembourg: Source not specified.

Poland: Sources not specified, other services: numbers from 2013.

Portugal: Sources not specified.

Romania: Sources not specified.

Slovenia: Sources not specified.

Spain: Sources not specified, beds for forensic patients provided in general psychiatric facilities and other services, numbers unknown.

Sweden: Specialized forensic facilities, forensic outpatient services: The Swedish National Forensic Psychiatric Register, beds in general psychiatric facilities provided in unknown number.

England: Specialized forensic facilities: Voluntary surveillance system of Royal College of Psychiatrists, no data on forensic outpatient services and prison services.

Switzerland: Number estimated by expert.

Countries not mentioned have failed to return information on the respective items.

The numbers reported were based on the concept of a “psychiatric bed or place” that had some “official” assignation or arrangement as being officially regarded as a forensic psychiatric bed or place. Despite that superficially more or less clear concept, a considerable proportion of the capacities either had to be estimated or were even totally unknown to a proportion of experts. No answers in the questionnaire for the respective services or items were left open in the boxes of Table 2. The footnotes of Table 2 show the data origins and areal ranges of figures if such information was provided.

**Figure 1:**
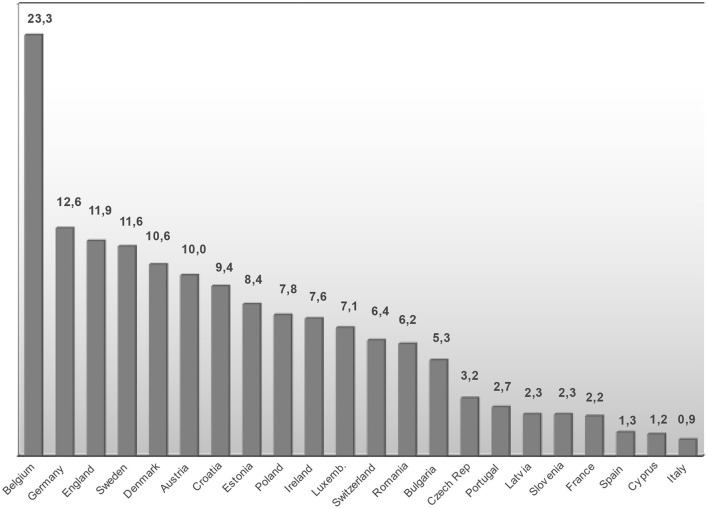
Forensic psychiatric beds per 100,000 population in European Union Member States in 2017 (forensic psychiatric beds in specialized forensic facilities, in general psychiatric facilities, in medical prison wards and in other services, excluding forensic psychiatric outpatient treatment capacities). Countries not mentioned have failed to return information on the respective items.

Despite these uncertainties, we summarized the number of forensic psychiatric beds in specialized forensic facilities, forensic psychiatric beds in general psychiatric facilities and forensic psychiatric beds in prison ward facilities and in other services to calculate the overall forensic psychiatric bed rates for every nation involved (columns 2–5 of Table 2). The population figures for the respective years were from Eurostat (19). Results are shown in Figure 1.

Even when taking into account the heterogeneity of this data, population-based rates reveal a highly variable pattern or service provision across Europe, ranging from 0.9 forensic psychiatric beds per 100,000 population in the reorganized forensic psychiatric service in Italy up to 23.3 in Belgium. Of all participating countries, Belgium provides the largest amount of forensic psychiatric beds that are located in general psychiatric hospitals (n= 1,068). Even when those beds were removed from the calculation, Belgium still had the largest per capita service among the included countries with 14.0 forensic psychiatric beds per 100,000 population.

There was almost no data on the national mean length of stay in forensic psychiatric services, although this information is essential for evaluating the effectiveness or outcome of the forensic psychiatric sector. When provided, mean length of stay data usually covered only selected services, and completely excluded patients in general psychiatric and prison service placements and thus were not reported here.

### Estimated prevalence rates of patients treated in forensic psychiatric services

The numbers of patients actually detained or treated in the available forensic beds were reported only from 13 out of the 22 participating countries. We used these figures as an estimate for the treated prevalence of forensic patients. Due to the greater uncertainty around the data on forensic psychiatric patients in general psychiatric hospitals and prison services, we calculated two prevalence rates, one for all facilities for which data was available and another covering only specialized forensic psychiatric facilities in 2017 (see Table 3).

**Table 3:** Estimates of treated prevalence of mentally ill offenders per 100,000 population in European Union Member States in 2017 (only forensic psychiatric inpatients, excluding forensic psychiatric patients in forensic psychiatric outpatient or other outpatient services; empty cells: unknown).

		**Number of forensic-psychiatric inpatients in**				**Rate per 100,000 population**	
	**Population (total number)**	**Specialized forensic facilities**	**General psychiatric facilities**	**Prison services**	**Other services**	**Only specialized forensic psychiatric facilities**	**All facilities**
Romania	19,638,300	1,503		3,044		7.7	23.3
Belgium	11,365,800	808	1,042	694		7.1	22.4
Germany	82,800,000	10,793				13.0	13.0
Estonia	1,315,600	148				11.2	11.2
Austria	8,772,900	440	210	230		5.0	10.0
Czech Republic	10,578,800	874	187			8.3	10.0
Poland	37,973,000	2,500		1,164		6.6	9.6
Sweden	9,995,200	933				9.3	9.3
Croatia	4 154,200	351	0	32		8.4	9.2
Slovenia	2,065,900	106				5.1	5.1
Latvia	1,950,100	45	29			2.3	3.8
Italy	60,589 400	599		328	1,800	0.9	2.9
Portugal	10,309,600	275				2.7	2.7

Germany: Data from 12 out of 16 federal states.

Poland: Specialized forensic facilities data estimated.

Czech Republic: General psychiatric facilities data from 2016.

Latvia: General psychiatric facilities and prison data only covering the region of the Capital of Riga.

Italy: “Other services” represents “controlled freedom” regimes.

Countries not mentioned have failed to return information on the respective items.

Considering all services that could detain or treat a mentally disordered offender, prevalence rates ranged from 2.7 per 100,000 population in Portugal up to 23.3 per 100,000 population in Romania. Looking only at specialized forensic psychiatric facilities, the lowest rate was 0.9 per 100,000 population in Italy and the highest was 13.0 per 100,000 population in Germany. However, this still underestimated the real prevalence in Germany, due to the lack of available data from 25 % of German Federal States.

### Indicators of incidence of forensic psychiatric patients

As accurate information on admissions and discharges to the various services was even rarer, we considered the annual numbers of defendants found not being guilty by reason of insanity as an estimate for the incidence of mentally ill offenders. That data was usually provided by the Ministries of Justice and more readily available. Table 4 shows the time series of these verdicts from 2010 onwards and the incidence rate per 100,000 population calculated for the most recent year available from 15 countries.

**Table 4:** Annual number of verdicts on persons not guilty for reason of insanity from 2010 to 2017 as an estimate for the incidence of mentally ill offenders in European Union Member States.

	**2010**	**2011**	**2012**	**2013**	**2014**	**2015**	**2016**	**2017**	**Population**	**Rate**
Denmark	746	788	781	770	788	694	649	655	5,748,800	11.4
Estonia		157	194	183	154	188	181	131	1,315,600	10.0
Poland								3,000	37,973,000	7.9
Czech Rep	501	498	503	528	581	691	564		10,578,800	5.3
Germany	3,271	3,308	3,243	3,272	3,256	3,278	3,370		82,800,000	4.1
Bulgaria			387	442	418	418	254	273	7,101,000	3.8
Slovenia				52	60	97	38	56	2,065,900	2.7
Sweden	234	293	219	284	261	296	293	273	9,995,200	2.7
Austria	89	110	86	92	68	97	137	155	8,772,900	1.8
Belgium	405	383	364	325	325	355	275	200	11,365,800	1.8
Croatia	102	137	79	70	51	43	53	32	4,154,200	0.8
Luxembourg	9	7	7	3	12	9	7	5	590,700	0.8
Finland	31	33	30	32	32	32	33	34	5,503,300	0.6
France	140								67,024,500	0.2
Ireland	3	1	2	5	6	5	16	7	4,774,800	0.1

Countries not mentioned have failed to return information on the items of this table (population: total number in 2017; rate: per 100,000 population most recent year; empty cells: no answer or unknown).

Again, the data suggest a wide variation in the annual incidence across Europe. Time series data indicate uneven trends of higher (e.g., Austria), lower (e.g., Belgium, Croatia, Bulgaria) or more or less stable incidence rates (e.g., Czech Republic, Denmark, Finland, Germany, Sweden) during the covered 8 year-period. Lowest estimates were 0.1 per 100,000 population in 2017 against 11.4 per 100,000 population as the highest rate in Denmark in 2017.

## Discussion

### Availability and validity of data

There is a serious gap in our knowledge about the basic characteristics and features of forensic psychiatric systems in European Union Member States. The common usage of the umbrella term “forensic psychiatry” tends to cover various understandings and practices. In many countries, even leading national experts or forensic psychiatrists in the field are unable to easily report valid basic numbers of the most essential indicators such as the number of forensic psychiatric beds, the incidence and prevalence of mentally disordered offenders or the mean length of stay in forensic psychiatric services. Outdated data or incomplete or time series (as in Table 4 in the case of France or Poland) suggest weak reporting standards regarding these issues in the health or judicial administration of these and other countries. Exact information on capacities of forensic outpatient services, forensic beds in general psychiatric hospitals and psychiatric beds in prison wards were particularly incomplete, or completely unknown even when it was reported that such services were in principle available.

### Strengths and limitations

From the perspective of validity and reliability, the inclusion of experts with varying background providing data from heterogenous non-standardized and in some cases not clearly defined sources is a serious methodological limitation. However, the current situation in European forensic psychiatry does not allow a better standardized data collection approach without risking to come up with even larger data gaps.

A considerable amount of informal, provisional, temporary or otherwise unregistered forensic beds in general psychiatric hospitals, outpatient services or medical prison wards cannot be ruled out in several countries. Crucial national outcome data, such as the rate of re-offending is totally lacking. These findings and conclusion are in line with the most recent study on the issue of Tomlin and colleagues who applied a similar approach and were confronted with similar obstacles (9).

The finding that national reporting systems on this crucial issue still seem to be grossly underdeveloped and that European registers are still missing is even more striking considering the repeated criticism of this failing by experts over the last decades.

### Heterogeneity of models and service provision

Despite the poor data-base it is evident that approaches and models of detaining and treating mentally disordered offenders are highly diverse across European Union Member States. The poorly standardized estimates presented here can only really be compared with an appreciation of the various legal and service provision frameworks that shape the national models (6, 20). However, we did not find any system characteristic or indicator from the questionnaire that would explain or justify a phenomenon as e.g., the much higher rate of forensic psychiatric capacities in Belgium. This great diversity of approaches prohibits a meaningful comparison of national forensic psychiatric systems merely on the basis of bed-rates or treated prevalence and makes it very difficult to draw any firm conclusions about the effectiveness of forensic psychiatric service models in Europe and worldwide.

### Consequences for health care policies and planning

The lack of clear data must be seen as a serious omission in a sector that is essential both for national psychiatric systems and for societies in general. Judicial frameworks that are overcome or incomplete or care concepts that are ineffective cannot be identified from the current scarce evidence. It also contributes to the fact that current forensic psychiatric treatment guidelines are rather general in nature (3).

This situation put European forensic psychiatry far behind the standards of related disciplines. Community psychiatry for instance has developed, discussed and agreed upon common concepts and guidelines and applies these in the improvement of national systems across Europe (21). The situation is inappropriate for a sector whose condition can be seen as a touchstone for a fair judicial framework, the high quality of mental health care provision and the overall level of humanity of any society. It seems absolutely essential to develop a strategy that would encompass over-arching concepts, harmonized regulations and practice guidelines for moving the field in that direction in future. To internationally agree on a common set of basic standardized indicators that are flexible enough for easy application across the various international forensic psychiatric systems is overdue. Mandatory national reporting systems need to be implemented.

This is first and foremost not a methodological challenge, but rather a political task to be tackled immediately. It is the task of the scientific community to combine their efforts by providing a binding methodological and research framework and increase pressure on national policy makers to implement it.

## Data availability statement

The data analyzed in this study is subject to the following licenses/restrictions: confidential. Requests to access these datasets should be directed to not applicable.

## Ethics statement

Written informed consent was obtained from the individual(s) for the publication of any potentially identifiable images or data included in this article.

## Author contributions

All authors listed have made a substantial, direct, and intellectual contribution to the work and approved it for publication.
